# Dose assessment in moving targets and organs at risk during carbon ion therapy for pancreatic cancer with respiratory gating

**DOI:** 10.1016/j.phro.2025.100775

**Published:** 2025-05-08

**Authors:** Christina Stengl, Jeppe B. Christensen, Iván D. Muñoz, Alexander Neuholz, Stephan Brons, Eduardo G. Yukihara, Jakob Liermann, Oliver Jäkel, José Vedelago

**Affiliations:** a Faculty of Medicine, University of Heidelberg, Im Neuenheimer Feld 672, Heidelberg 69120, Germany; b Division of Medical Physics in Radiation Oncology, German Cancer Research Center (DKFZ), Im Neuenheimer Feld 280, Heidelberg 69120, Germany; c Heidelberg Institute for Radiation Oncology (HIRO) and National Center for Radiation Research in Oncology (NCRO), Im Neuenheimer Feld 280, Heidelberg 69120, Germany; d Department of Radiation Safety and Security, Paul Scherrer Institute (PSI), Forschungsstrasse 111, Villingen PSI, 5232, Switzerland; e Department for Physics and Astronomy, University of Heidelberg, Im Neuenheimer Feld 226, Heidelberg 69120, Germany; f Heidelberg Ion Beam Therapy Center (HIT), Heidelberg University Hospital (UKHD), Im Neuenheimer Feld 450, Heidelberg 69120, Germany; g Department of Radiation Oncology, Heidelberg University Hospital (UKHD), Im Neuenheimer Feld 400, Heidelberg 69120, Germany

**Keywords:** Anthropomorphic phantom, Respiratory gating, Carbon ion radiotherapy, Optically stimulated luminescence detectors (OSLDs)

## Abstract

**Background and purpose:**

Carbon ion radiotherapy (CIRT) has demonstrated promising treatment outcomes for pancreatic cancer. However, breathing-induced organ motion can compromise the efficacy of the treatment, leading to under- or over-dosage within the target and organs at risk (OARs). In this work, the dose during CIRT was simultaneously measured at the target and OARs using an anthropomorphic phantom to evaluate the effectiveness of respiratory gating for compensating breathing motion.

**Materials and methods:**

The Pancreas Phantom for Ion beam Therapy (PPIeT) was irradiated with carbon ions. The phantom features a pancreas with a virtual tumour and OARs including a duodenum, kidneys, a spine and a spinal cord. Breathing-induced organ motion was imitated with amplitudes of 0 mm (control), 5 mm, 10 mm and 20 mm while irradiating with and without gating. Dose measurements were performed using an ionisation chamber and passive detectors.

**Results:**

The prescribed uniform dose of 1.37 Gy in the virtual tumour was experimentally validated for the control. Breathing-induced motion of 20 mm led to a 75 % dose coverage at the target improving to 91 % with gating. For the OARs, the mean dose varied according to the organ, with gating showing no significant differences.

**Conclusions:**

Accurate CIRT dosimetry with variable breathing-induced motions can be conducted with PPIeT for a pancreatic tumour and the OARs. Gating mitigated the effects of breathing-induced motion in the tumour.

## Highlights

• Breathing-induced organ motion reduces target dose in pancreatic cancer treatment.
• Respiratory gating mitigates the dose reduction in the pancreas tumour.
• Respiratory gating does not significantly affect the dose in the organs at risk.

## 1 Introduction

In recent decades, carbon ion radiotherapy (CIRT) has been demonstrated to effectively treat pancreatic cancer compared to conventional radiotherapy [1]. In CIRT, the spatial accuracy of the ion beam is exploited enabling high dose conformity in the tumour and increased healthy tissue sparing [2]. Despite its demonstrated efficacy, CIRT for pancreatic cancer patients is still in its infancy and is currently offered at only a few specialised centres globally [3]. Most of the clinical experience comes from Japan but treatment delivery differs compared to European facilities [1], [4], [5], [6].

Breathing-induced organ motion, particularly in the context of ion treatment, can result in under-dosage of the tumour and over-dosage of organs at risk (OARs) [7], [8], [9], [10]. Motion management strategies have been increasingly used in radiotherapy, including breath-hold techniques, body compression and respiratory gating [11], [12], [13], [14]. The primary drawbacks of breath-hold and abdominal compression are the compatibility with co-malignancies and the discomfort to the patient. Instead, gating enables the patient to breathe normally during treatment, compensating for abdominal motion effects by synchronising the beam delivery with the breathing pattern [12]. However, the potential benefits of gating for CIRT on pancreatic cancer remain uncertain and the studies in this context are sparse [9], [15]. To ensure the precision and safety of treatment delivery it is essential to accurately estimate the delivered dose before translating new techniques into clinical practice. Therefore, phantoms are crucial in pre-clinical phases. While many phantoms have geometric shapes that do not resemble the human body, anthropomorphic phantoms replicate a more human-like environment, allowing for realistic dose assessment [16], [17], [18]. Advancements in anthropomorphic phantoms include breathing motion for tumours and OARs [19], [20], [21], [22]. A dedicated phantom was developed for this purpose, conducting dosimetry on carbon ion radiotherapy and later studying breathing motion during carbon ion mini-beam radiotherapy [23], [24].

This study aims to assess the impact of breathing motion on the dose distribution during pancreatic cancer CIRT by simultaneously measuring the dose in the target and multiple OARs, including the duodenum, kidneys, spine, and spinal cord. Using an anthropomorphic phantom with complex organ motion, the study evaluates the feasibility and the effectiveness of gating in mitigating motion-related dose variations across the target and the OARs.

## 2 Materials and methods

### 2.1 Anthropomorphic phantom with breathing motion feature

For the dose assessment of pancreatic cancer with CIRT, the anthropomorphic abdominal Pancreas Phantom for Ion beam Therapy (PPIeT) with breathing motion feature was used as previously described [23]. PPIeT consists of a propylene container with a flexible abdominal wall that allows breathing-induced organ motion, including a dosimetric insert of 25.6  mm × 37.6  mm × 23.3  mm in the pancreas head considered as the virtual tumour, along with a duodenum, two kidneys, a segment of the spine and the spinal cord as OARs. The organs were embedded in 0.25 % w/w superabsorber-water mixture (Schauch, Germany). A motor-controlled actuator was connected to the flexible diaphragm to induce breathing motion, transmitting the motion to the organs and the abdominal wall. The motor was controlled by a PLC CX5020 (Beckhoff, Germany) using TwinCat Version 3 (TcXaeShell Version 15.0.28010.2050 D15.8, Beckhoff, Germany) enabling arbitrary breathing patterns. For this study sinusoidal breathing curves with a phase of 7 s were used to induce organ motion with amplitudes of 5 mm, 10 mm and 20 mm in caudal-cranial direction [23].

### 2.2 Dosimetry within organs

To measure doses inside the organs in PPIeT, special inserts were 3D-printed in Veroclear (Stratasys, Israel) to accommodate either an ionisation chamber (IC) (PTW PinPoint 31015, Germany) or optically stimulated luminescence detectors (OSLDs) (Supplementary Table S1) [23]. During motion, the detectors were fixed in the target and therefore followed the target’s exact motion. Both detector types were employed in the virtual tumour (Supplementary Fig. S1, S2). For dosimetry in the OARs, OSLDs were placed in the organ inserts (Supplementary Figs. S4-S6). The OSLDs were cut from a *∼* 100 µm thick film to form discs with a diameter of 4 mm [25]. They were placed in a 2D array in each organ insert to measure the 2D dose distribution. Their ability to assess the linear energy transfer using the different emission bands in the Al_2_O_3_:C-based OSLDs enabled a quenching correction, and thus the applicability for carbon ion dosimetry [26], [27], [28], [29], [30].

### 2.3 Carbon ion radiotherapy treatment

A planning CT was performed with the Definition Flash CT scanner (Siemens Healthineers, Germany), using an abdominal scan with 480 mA and 120 kV as previously reported [23]. The CIRT plan with 4 Gy (RBE) biological dose^1^ was computed using the local effect model (LEM) I model with RayStation (Version 11B (12.0.0.932), RaySearch Laboratories, Sweden). This corresponds to a mean of 1.37 Gy physical dose at the virtual tumour.

**Fig. 1:**
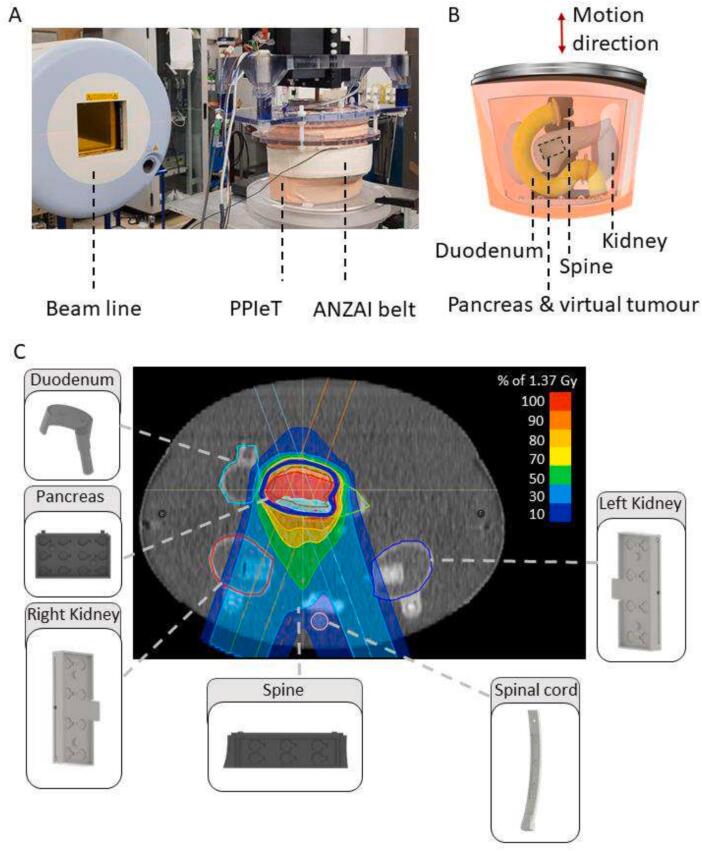
Experimental setup displaying the anthropomorphic phantom PPIeT in up- right position in front of the beamline. The phantom is equipped with the ANZAI belt for gating (A). Schematic of PPIeT with organs and virtual tumour (dashed box). The motion direction is indicated by the red arrow (B). Treatment plan of the physical dose with the blue line delineating the PTV. The dose overshoot is below 4 %. Each organ insert is shown from inside illustrating the OSLD positions. The dashed grey lines show the positions of the insert (C).

Irradiations were performed at the Heidelberg Ion Beam Therapy Centre (HIT; Heidelberg, Germany) using a carbon ion pencil beam scanning technique, with and without gating. The static measurements were conducted without motion input in the same configuration as the planning CT was done. For the three different motion amplitudes, breathing motion was applied using the motor-controlled actuator. During gating the beam was synchronized with the gating window using the ANZAI belt system AZ-733VI (Anzai Medical Co. Ltd., Japan) ensuring irradiation occurred during 50 % of the breathing cycle [32]. A picture of the experimental setup is shown in Fig. 1. The phantom was positioned in an in-house fabricated rotational plate to allow irradiation from the different angles with the horizontal beam line.

For each motion amplitude, four independent measurements were conducted with the IC and three independent measurements were performed with the OSLDs by exchanging the inserts after each treatment plan irradiation. For each position, the standard deviation of these repetitions is the Type A uncertainty [33]. All the sources of uncertainty are listed in Supplementary Table S2. The results are reported as the mean values ± the combined uncertainty with a coverage factor of *k* = 1. To compare the OSLD and IC measurements at the virtual tumour positions, the two OSLD measurements at the IC position in the middle of the insert were averaged (Supplementary Fig. S1, S2). Statistical significance was assessed using a two-way ANOVA, and significance was defined as *p* *<* 0.05.

## 3 Results

### 3.1 Partial restoration of target dose with respiratory gating

**Fig. 2:**
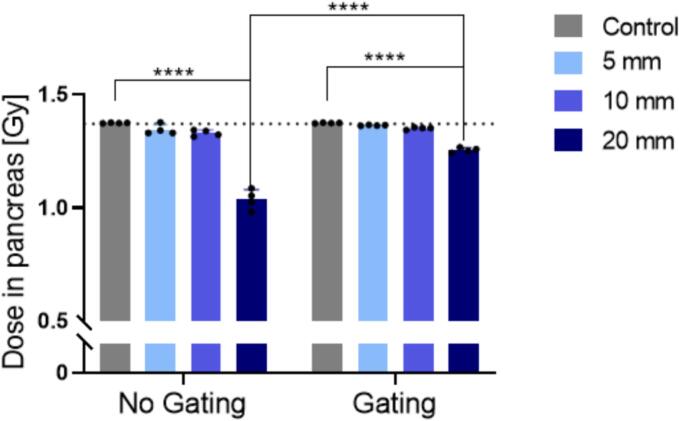
Target dose measured with an IC positioned within the virtual tumour. CIRT was performed with and without gating for the control and different breathing motions. The dotted line indicates the expected target dose of the treatment plan. The black dots indicate single measurements and the error bars represent their standard deviation. Significance: **** *p* *<* 0.0001.

A first evaluation of the motion effect in the virtual tumour during CIRT was done with the IC, measuring the dose with and without gating. The measurement with the IC for the static condition, named control, yielded (1.374 ± 0.038) Gy, in agreement with the calculated dose of the treatment plan (Fig. 2). Without gating, organ motions of 5 mm and 10 mm exhibited no significant reduction in the measured target dose. For 20 mm motion, however, (1.036 ± 0.053) Gy was measured, representing 75 % of the target dose. The use of gating consistently decreased the difference between measured and planned dose. 20 mm motion with gating significantly mitigated the underdosage, yielding a target dose of (1.254 ± 0.037) Gy, representing 91 % of the target dose. Gating reduced the standard deviation for all motion amplitudes.

The dose values used to validate the OSLD dosimetry were in agreement with a 2 % mean deviation under all conditions compared to the IC (Supplementary Fig. S2, S3). Therefore, OSLDs were used to measure the dose in the OARS.

**Fig. 3:**
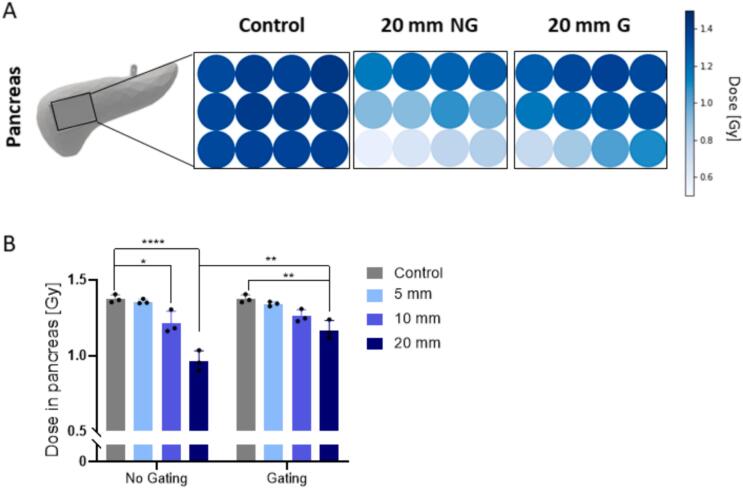
2D dose values for the virtual tumour measured with OSLDs for the control, the 20  mm motion without gating (NG) and with gating (G). The 2D colour representation in these figures does not reflect the actual spacing between the OSLDs. The colour intensity represents the dose value in Gy (A). Mean values over the whole virtual tumour for the different motions (B). Significance: * *p* *<* 0.05; ** *p* *<* 0.005; **** *p* *<* 0.0001.

The 2D dose measured with the OSLDs without motion was uniformly distributed over the whole virtual tumour with a deviation of less than 2 % (Fig. 3A, Supplementary Fig. S1). However, 20 mm motion led to a dose gradient within the PTV, decreasing from (1.27 ± 0.12) Gy at the top right of the virtual tumour to (0.584 ± 0.096) Gy at its bottom left (Supplementary Table S3). Gating led to a reduction of the gradient by 12 % while increasing the overall dose in the tumour. The mean dose in the virtual tumour of the control was (1.38 ± 0.11) Gy (Fig. 3B). 5 mm motion did not reduce the mean dose significantly. Motion of 10 mm and 20 mm led to a significant mean dose reduction representing 88 % (*p* = 0.0156) and 70 % (*p* *<* 0.0001) of the target dose, respectively. The application of gating mitigated the dose reduction in the virtual tumour, resulting in no significant differences for 10 mm motion compared to the control. However, even with gating a significant underdosage for 20 mm motion was observed with a mean target dose of (1.16 ± 0.22) Gy, representing 84 % of the target dose.

### 3.2 Motion impact on the organs at risk

**Fig. 4:**
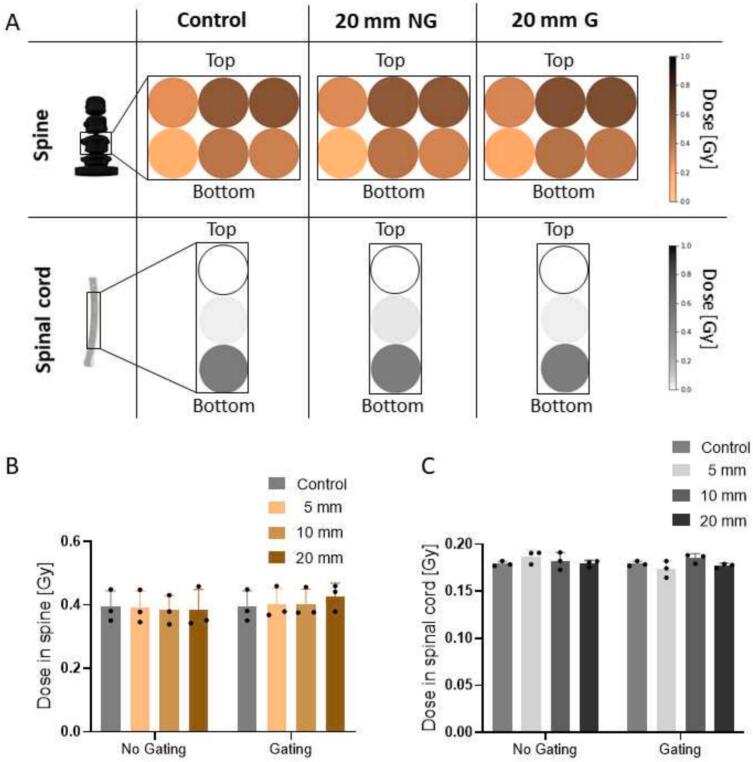
2D dose values of the spine and the spinal cord for control, 20 mm without gating (NG) and with gating (G). The colour intensity represents the dose value in Gy (A). Mean values of the spine insert (B) and spinal cord insert (C) for the different motions.

The dose in the immobile OARs (spine and spinal cord) remained unaffected by motion for all motion amplitudes (Fig. 4, Supplementary Fig. S4). Within the spine, the dose ranged from (0.148 ± 0.042) Gy to (0.573 ± 0.073) Gy, whereas in the spinal cord, it ranged from 0 Gy to (0.513 ± 0.041) Gy (Supplementary Table S4).

**Fig. 5:**
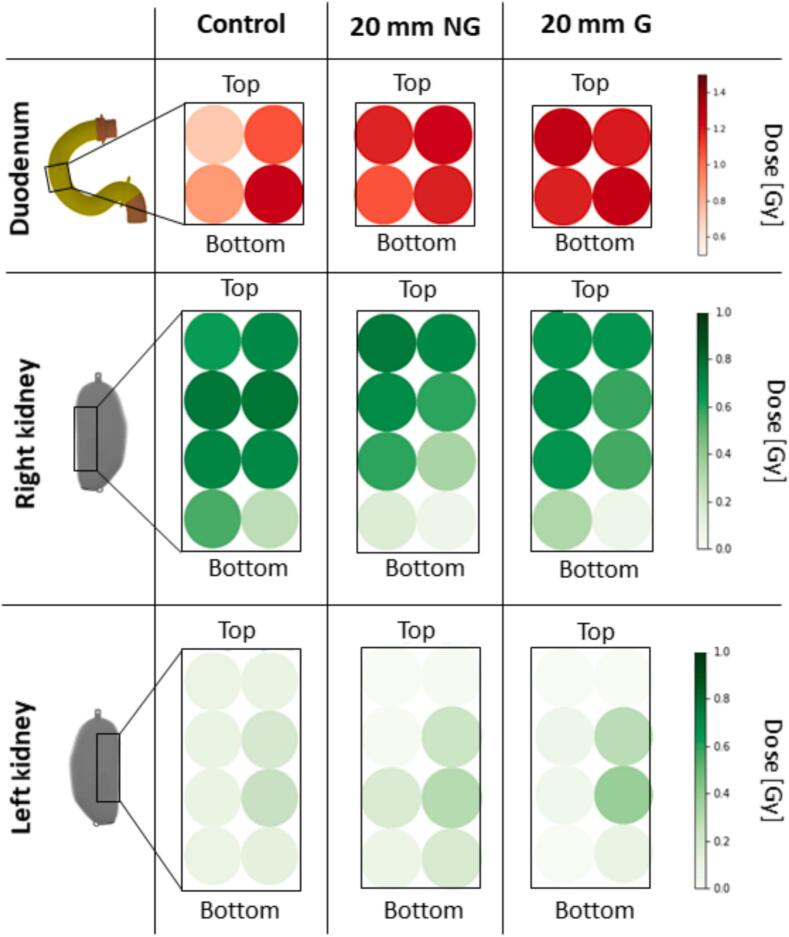
2D dose values for the duodenum (A), the right kidney (B), and the left kidney (C) for control, 20 mm motion without gating (NG) and 20 mm motion with gating (G).

**Fig. 6:**
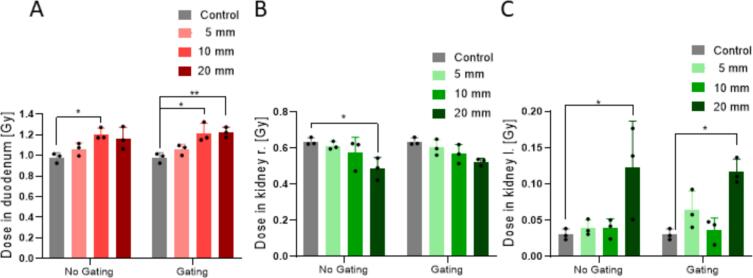
Mean values of the dose in the duodenum insert (A), the right kidney insert (B) and the left kidney insert (C) for the different motions. Significance: * *p* < 0.05; ** *p* < 0.005.

The 2D dose evaluation in the duodenum showed an inhomogeneous distribution for the control (Fig. 5, Supplementary Fig. S5). A minimum dose of (0.72 ± 0.12) Gy and a maximum dose of (1.24 ± 0.13) Gy was measured (Supplementary Table S4). With a motion amplitude of only 5 mm, the minimum dose increased by 32 %, while 20 mm motion resulted in a 64 % increase. On average, the duodenum received (0.978 ± 0.087) Gy for the control, which increased by 19 % for the 20 mm motion condition (Fig. 6). Gating did not reduce the total dose in any motion condition but reduced the relative uncertainty for 20 mm motion by 26 %.

Within the right kidney, a decrease in dose was observed for 20 mm motion, particularly at the bottom parts, which received only 29 % (right) and 23 % (left) of the control dose. In contrast, the dose to the left kidney increased across all positions, with the maximum dose increasing more than two times for 20 mm motion (Fig. 5, Supplementary Fig. S6). The average dose to the right kidney was (0.64 ± 0.18) Gy for the control, and decreased significantly by 24 % for 20 mm motion (*p* = 0.0211). A significant increase of more than four times in the average dose of the left kidney was observed (*p* = 0.0135). Gating did not affect the average dose values when comparing each motion without gating. For 20  mm motion, gating reduced the relative uncertainty by 72 % in the left kidney.

## 4 Discussion

Ensuring precise and safe treatment delivery is crucial in radiotherapy. In the case of pancreatic cancer, studies on respiratory gating during CIRT are limited. This work evaluates the potential benefits of gating in CIRT for pancreatic cancer, focusing on its effects on the absorbed dose at the target and OARs simultaneously. The obtained results for increasing breathing-induced motion indicate a target dose reduction. Gating helps mitigate dose reduction in the tumour without significantly affecting the OARs.

The dose was assessed using an IC for dose verification in the pancreas, resulting in a combined uncertainty similar to previous studies with protons [34]. Additionally, OSLDs were used to measured the dose in all organs. Their small size allowed to position several detectors in a 2D array within the organ inserts, enabling a 2D dose distribution estimation. Using this 2D array, it was possible to measure a larger area compared to the single measurements from the IC, and to identify dose variations across the measured area. That is why for a motion amplitude of 5 mm no significant differences in the virtual tumour dose were evident for both IC and OSLDs. At 10 mm motion, a significant dose reduction was detected only by the OSLDs, as they covered the entire tumour, unlike the IC, which only measured within the centre. However, the IC was getting close to the penumbra of the planned target and therefore a small dose decrease of 3 % was found also increasing the uncertainty. For 20 mm motion, both detector types measured a significant dose reduction in the virtual tumour.

Breathing-induced organ motion of 5 mm or less does not significantly affect the treatment dose, regardless of the dosimeter used. This finding verifies the commonly suggested threshold of 5 mm for not employing motion mitigation techniques [14], [35], [36], [37]. For larger motion amplitudes, a previous study using the same phantom for carbon ion mini-beam irradiation also reported decreasing mean dose values at the virtual tumour for increasing motion amplitudes [24]. In a study with another phantom, a motion amplitude of only 6 mm significantly disturbed carbon ion dose distributions, as measured at multiple positions within the target using various ICs [38]. This finding highlights the importance of evaluating 2D dose distributions, as demonstrated with PPIeT, where a 10 mm motion resulted also in significant dose differences within the target. For the OARs, 10 mm motion did not affect the dose significantly, except for the duodenum located directly next to the pancreas. For the largest motion used in PPIeT, namely the 20 mm motion, an increased dose deviation was found in the target, showing consistency with previous CIRT studies [38].

The efficacy of gating has been a subject of ongoing debate for an extended period [9], [15], [39], [40], [41]. One study reported minimal OAR dose reduction with gating, insufficient to justify longer treatment times [15], while another study observed improved target dose homogeneity [9]. Nevertheless, 85 % of the clinical centres worldwide use either rescanning or active respiratory motion management systems [42]. For example, a technique was presented in which a set of motion-phase specific plans are delivered [41]. However, systematic studies remain limited, and further improvements are needed [42].

In contrast, the approach presented here using PPIeT enabled simultaneous target and OARs dose measurement within an anthropomorphic phantom. While for 5 mm motion no gating is necessary, 20 mm motion with gating significantly improved dose homogeneity within the tumour, along with a partially recovered mean dose. Regarding the OARs, for the duodenum and the left kidney the dose increased upon motion and gating did not alter this increment but improved dose variability. Interestingly, a dose reduction was observed in the right kidney with motion, explained by the organ moving out of the beam. Thus, the impact of breathing-induced motion in the OARs dose cannot be generalised and must be analysed individually for each organ and each patient’s anatomy. This highlights the importance of implementing 4D dose calculations in the clinic to account for patient-specific motion effects [42].

A more comprehensive investigation could include smaller increments in the motion amplitudes with values between and beyond the ones used. Furthermore, patient-specific breathing amplitudes as well as irregular breathing patterns could be tested by using the presented workflow, keeping in mind that the anatomy is the one of the phantom.

In conclusion, by employing an anthropomorphic phantom for dosimetry in carbon ion radiotherapy, this study underscores the importance of tailoring the decision to use gating for individual patient breathing. An ionisation chamber in combination with passive detectors allowed for comprehensive dose measurement within the pancreas virtual tumour and simultaneously in the organs at risk. Based on the ionization chamber results for 20 mm motion, gating improved the dose coverage at the target from 75 % to 91 %. Furthermore, based on the passive detector results, the dose at the organs at risk was not affected.

## CRediT authorship contribution statement

**Christina Stengl:** Conceptualization, Data curation, Formal analysis, Investigation, Methodology, Software, Visualization, Writing – original draft, Writing – review & editing. **Jeppe B. Christensen:** Conceptualization, Investigation, Methodology, Data curation, Software, Writing – review & editing. **Iván D. Muñoz:** Investigation, Writing – review & editing. **Alexander Neuholz:** Methodology, Writing – review & editing. **Stephan Brons:** Methodology, Writing – review & editing. **Eduardo G. Yukihara:** Writing – review & editing. **Jakob Liermann:** Conceptualization, Writing – review & editing. **Oliver Jäkel:** Conceptualization, Supervision. **José Vedelago:** Conceptualization, Supervision, Writing – review & editing.

## Declaration of competing interest

The authors declare that they have no known competing financial interests or personal relationships that could have appeared to influence the work reported in this paper.

## Footnotes

1 ^1^The Gy (RBE) refers to the ICRU 93 definition [31].
