# Supplementary material for: Dose assessment in moving targets and organs at risk during carbon ion therapy for pancreatic cancer with respiratory gating
Source: Phys Imaging Radiat Oncol. 2025 May 8;34:100775. doi: 10.1016/j.phro.2025.100775 (PMC12141065; doi:10.1016/j.phro.2025.100775)
Supplement: Supplementary Data 1 [file mmc1.pdf]

# Supplementary material

## Dose assessment in moving targets and organs at risk during carbon ion therapy for pancreatic cancer with respiratory gating

Christina Stengl, Jeppe B. Christensen, Iván D. Muñoz, Alexander Neuholz, Stephan Brons, Eduardo Yukihara, Jakob Liermann, Oliver Jäkel, José Vedelago

**Supplementary Table S1**: Insert sizes of each organ and corresponding number of OSLDs used in the study.

| **Organ** | **Insert size [$mm^3$]** | **Number of OSLDs** |
|---|---|---|
| Pancreas | 25.6 × 37.6 × 23.3 | 12 |
| Kidney | 20.0 × 50.0 × 8.6 | 8 |
| Spine | 14.8 × 42.9 × 11.5 | 6 |
| Spinal cord | 10.0 × 100.6 × 7.0 | 3 |

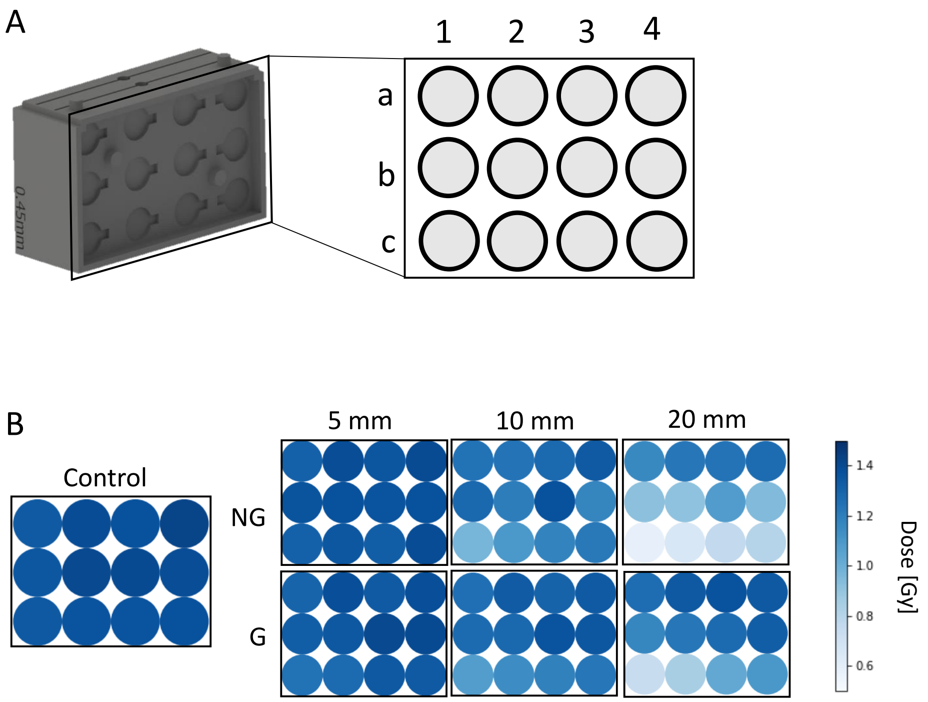


**Supplementary Figure S1**: 3D-printed pancreas target insert with a total of 12 positions for OSLDs with an arrangement of the OSLDs in rows a to c and columns 1 to 4 (A). Dose values of OSLDs for control and the three different motion amplitudes with and without gating. The 2D colour representation in these figures does not reflect the actual spacing between the OSLDs. The colour intensity represents the dose value in Gy (B).

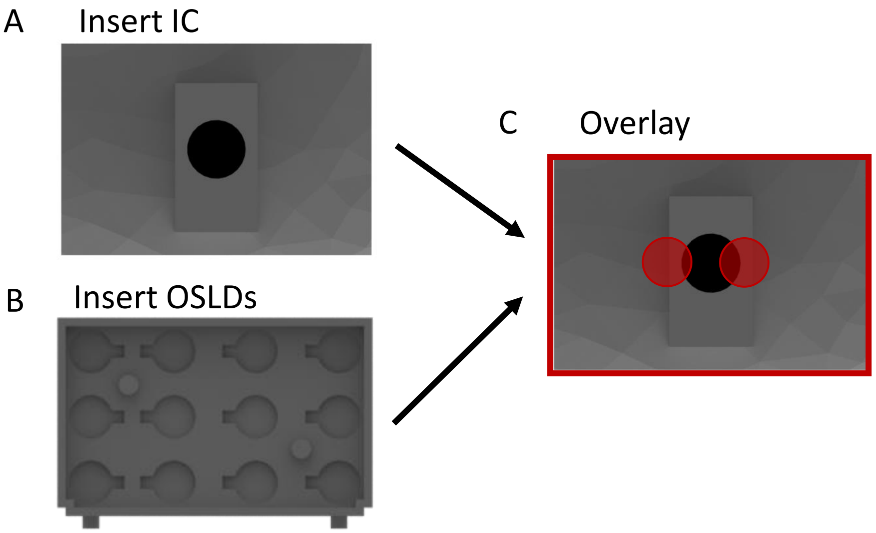


**Supplementary Figure S2**: 3D-printed pancreas inserts were created to accommodate the IC (A) or OSLDs (B). In the overlay of the inserts for IC and OSLDs, positions b2 and b3 of the OSLDs overlap with the position of the IC (C). Therefore, these two OSLDs were used for comparing measurements between the IC and OSLDs.

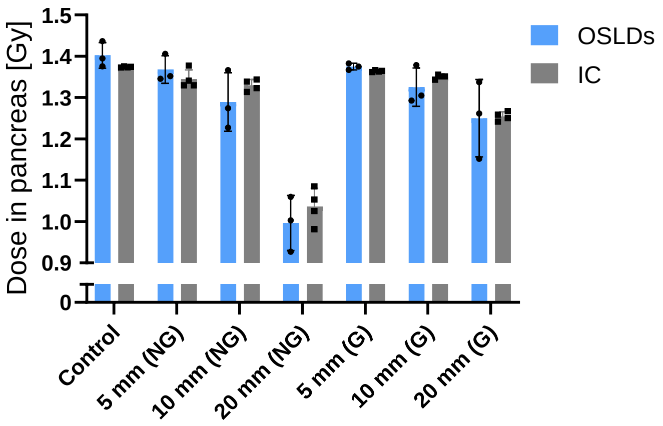


**Supplementary Figure S3**: Comparison between dose measured by OSLDs and IC in the pancreas virtual tumor for control and motion without gating (NG) and gating (G). The OSLD dose was calculated by taking the mean value of the OSLDs in positions b2 and b3 as explained in Supplementary Figure S2. Points indicate three independent measurements.

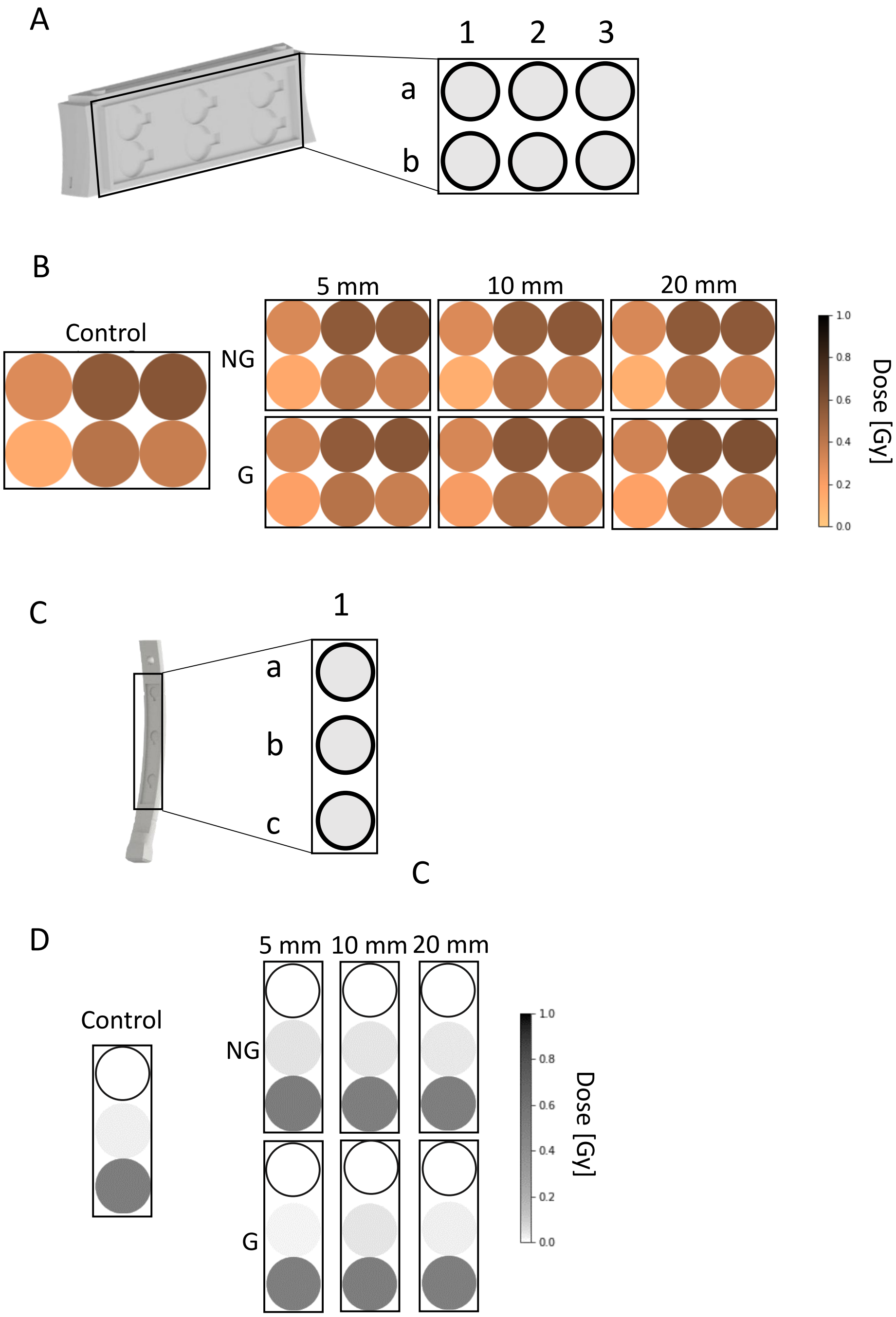


**Supplementary Figure S4**: 3D-printed spine insert with a total of 6 OSLDs positions arranged in rows a to b and columns 1 to 3 (A). Dose values of OSLDs for control and the three different motion amplitudes with and without gating (B). Insert of spinal cord with a total of 3 OSLDs positions arranged in rows a to c and column 1 (C). Dose values of OSLDs for control and the three different motion amplitudes with and without gating (D).

**Supplementary Table S2**: Uncertainty budget. For the OSLDs, the Type A uncertainty resulting from three independent measurements is given in Supplementary Tables S2-S3 for each position.

| **Uncertainty source** | **Type** | **Contribution [%]** | **Reference** |
|---|---|---|---|
| IC calibration | B | 1.1 | * |
| OSLD calibration | B | 7.0 | [1] |
| Positioning | B | 2.5 | [2] |
| Dose delivery | B | 0.7 | [3] |
| IC combined uncertainty | B | 2.8 | |
| OSLD combined uncertainty | B | 7.5 | |
| IC reproducibility | A | 0.1 to 4.3 | Figure 2 |
| OSLD reproducibility | A | 1.1 to 52.9 | Figures 3, 4, 6 |
| IC combined standard uncertainty | A+B | 2.8 to 5.1 | |
| OSLD combined standard uncertainty | A+B | 7.6 to 53.4 | |

*Calibration certificate from the manufacturer.

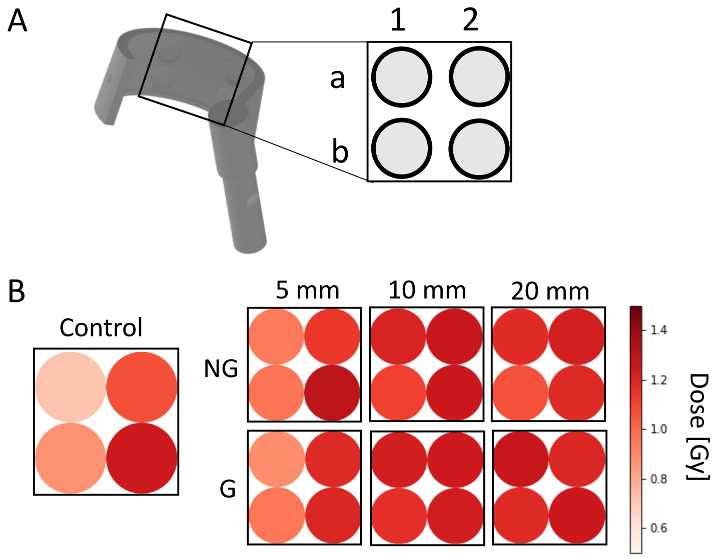


**Supplementary Figure S5**: 3D-printed duodenum insert with a total of 4 OSLDs positions arranged in rows a to b and columns 1 to 2 (A). Dose values of OSLDs for control and the three different motion amplitudes with and without gating (B).

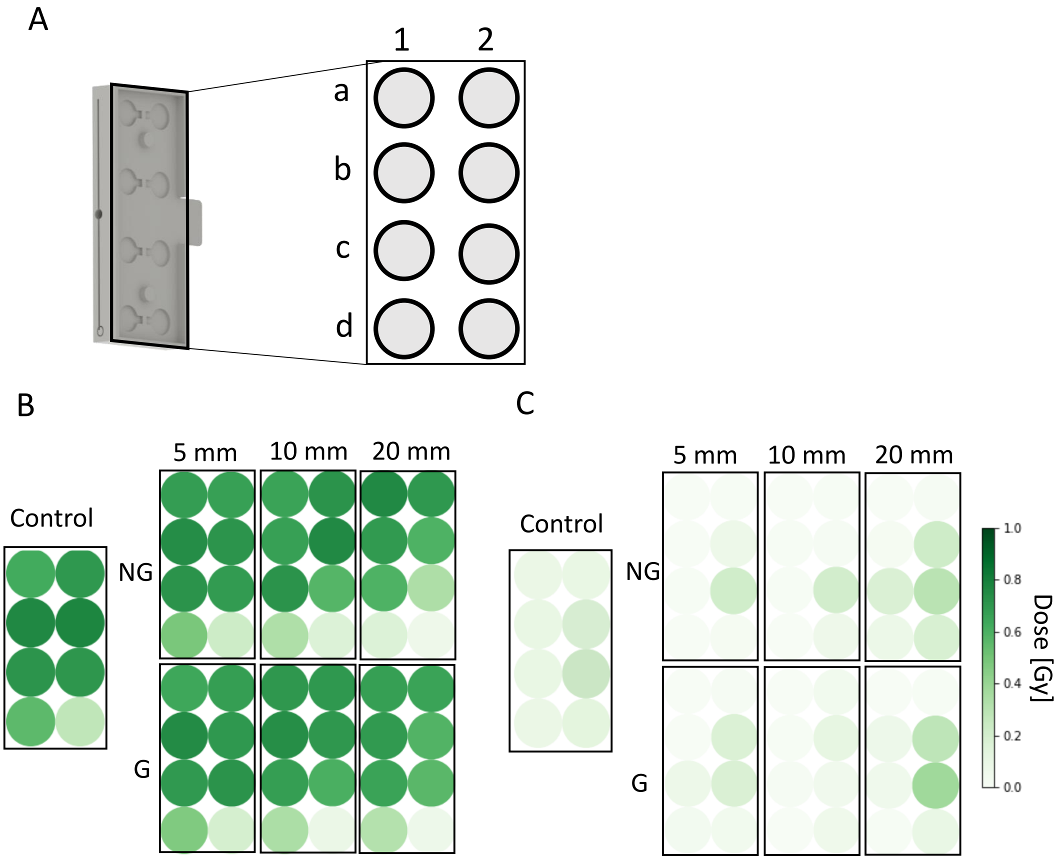


**Supplementary Figure S6**: 3D-printed right kidney insert with a total of 8 OSLDs positions arranged in rows a to d and columns 1 to 2 (A). The insert for the left kidney has a total of 8 OSLDs positions with the same distribution as for the right kidney. Dose values of OSLDs for control and the three different motion amplitudes with and without gating in the right (B) and left (C) kidney.

**Supplementary Table S3**: Mean value and standard deviation of the measured doses with the OSLDs at each position in the pancreas for the control, the different motion amplitudes and the different motion amplitudes with gating. The reported values are in Gy. The number in parentheses is the standard deviation of the three independent measurements and refers to the last two digits of the respective mean value.

| **Position** | **Control** | **5 mm** | **10 mm** | **20 mm** | **5 mm Gating** | **10 mm Gating** | **20 mm Gating** |
|---|---|---|---|---|---|---|---|
| a1 | 1.338(39) | 1.312(67) | 1.25(16) | 1.16(22) | 1.298(36) | 1.256(55) | 1.264(72) |
| a2 | 1.392(31) | 1.388(82) | 1.24(14) | 1.23(12) | 1.3854(27) | 1.333(42) | 1.343(65) |
| a3 | 1.373(18) | 1.352(35) | 1.28(17) | 1.24(15) | 1.338(16) | 1.301(48) | 1.370(29) |
| a4 | 1.425(56) | 1.395(33) | 1.339(16) | 1.268(67) | 1.385(35) | 1.337(42) | 1.340(62) |
| b1 | 1.348(33) | 1.361(47) | 1.28(14) | 0.921(66) | 1.324(21) | 1.272(10) | 1.172(77) |
| b2 | 1.404(37) | 1.3724(81) | 1.21(14) | 0.91(14) | 1.347(25) | 1.284(39) | 1.23(11) |
| b3 | 1.401(27) | 1.364(60) | 1.373(30) | 1.08(11) | 1.4034(99) | 1.367(55) | 1.270(97) |
| b4 | 1.389(38) | 1.371(60) | 1.18(21) | 0.95(24) | 1.399(25) | 1.349(71) | 1.324(61) |
| c1 | 1.3424(88) | 1.312(16) | 0.97(16) | 0.583(85) | 1.24(72) | 1.077(70) | 0.74(13) |
| c2 | 1.364(21) | 1.350(21) | 1.10(11) | 0.66(10) | 1.28(37) | 1.144((71) | 0.84(12) |
| c3 | 1.365(56) | 1.322(57) | 1.18(18) | 0.765(79) | 1.342(26) | 1.188(76) | 1.02(12) |
| c4 | 1.374(36) | 1.393(17) | 1.22(17) | 0.80(11) | 1.338(45) | 1.229(74) | 1.10(15) |

**Supplementary Table S4**: Mean value and standard deviation of the measured doses with the OSLDs at each position in the spine, the spinal cord, the duodenum, the right kidney and the left kidney for the control condition and the different motion amplitudes with and without gating. The reported values are in Gy. The number in parentheses is the standard deviation of the three independent measurements and refers to the last two digits of the respective mean value.

| **Organ** | **Position** | **Control** | **5 mm** | **10 mm** | **20 mm** | **5 mm Gating** | **10 mm Gating** | **20 mm Gating** |
|---|---|---|---|---|---|---|---|---|
| Spine | a1 | 0.301(50) | 0.316(38) | 0.308(41) | 0.317(63) | 0.320(44) | 0.323(50) | 0.342(45) |
| | a2 | 0.550(52) | 0.550(44) | 0.526(61) | 0.551(57) | 0.540(74) | 0.553(48) | 0.588(20) |
| | a3 | 0.574(59) | 0.549(69) | 0.558(61) | 0.551(72) | 0.570(56) | 0.551(66) | 0.601(48) |
| | b1 | 0.148(40) | 0.158(36) | 0.1269(75) | 0.12(11) | 0.199(49) | 0.215(67) | 0.192(47) |
| | b2 | 0.420(48) | 0.421(57) | 0.418(47) | 0.422(64) | 0.422(64) | 0.424(66) | 0.434(59) |
| | b3 | 0.371(68) | 0.308(41) | 0.364(85) | 0.344(74) | 0.366(67) | 0.354(59) | 0.406(62) |
| Spinal cord | a1 | * | * | * | * | * | * | * |
| | b1 | 0.024(13) | 0.044(28) | 0.041(15) | 0.037(36) | 0.016(17) | 0.043(25) | 0.026(33) |
| | c1 | 0.513(15) | 0.516(12) | 0.505(22) | 0.502(28) | 0.505(40) | 0.512(12) | 0.505(40) |
| Duodenum | a1 | 0.72(11) | 0.950(17) | 1.210(87) | 1.18(20) | 0.890(92) | 1.23(15) | 1.2656(64) |
| | a2 | 1.071(35) | 1.137(64) | 1.26(15) | 1.22(21) | 1.185(52) | 1.239(59) | 1.198(99) |
| | b1 | 0.876(80) | 0.968(93) | 1.11(11) | 1.07(13) | 0.958(55) | 1.17(25) | 1.18(13) |
| | b2 | 1.244(91) | 1.29(16) | 1.25(10) | 1.187(83) | 1.197(34) | 1.233(62) | 1.253(92) |
| Right kidney | a1 | 0.622(78) | 0.678(39) | 0.654(63) | 0.756(23) | 0.639(53) | 0.702(42) | 0.67(12) |
| | a2 | 0.700(22) | 0.666(51) | 0.718(18) | 0.699(60) | 0.675(21) | 0.6986(41) | 0.654(20) |
| | b1 | 0.758(59) | 0.742(75) | 0.67(12) | 0.69(19) | 0.750(68) | 0.74(10) | 0.69(14) |
| | b2 | 0.770(25) | 0.713(96) | 0.756(58) | 0.59(25) | 0.70(11) | 0.70(17) | 0.58(20) |
| | c1 | 0.713(54) | 0.718(79) | 0.72(10) | 0.592(88) | 0.692(79) | 0.674(88) | 0.653(13) |
| | c2 | 0.706(32) | 0.686(57) | 0.58(19) | 0.334(67) | 0.717(69) | 0.602(72) | 0.57(13) |
| | d1 | 0.56(11) | 0.48(15) | 0.33(22) | 0.162(37) | 0.47(20) | 0.34(13) | 0.32(19) |
| | d2 | 0.27(16) | 0.22(11) | 0.162(65) | 0.064(43) | 0.19(12) | 0.087(88) | 0.068(16) |
| Left kidney | a1 | * | 0.0020(34) | * | 0.076(67) | 0.032(55) | 0.0022(37) | 0.0115(97) |
| | a2 | 0.027(11) | 0.0168(68) | 0.064(30) | 0.18(13) | 0.045(46) | 0.060(82) | 0.096(46) |
| | b1 | 0.0049(86) | 0.0040(34) | 0.0091(87) | 0.17(16) | 0.07(11) | 0.014(14) | 0.056(40) |
| | b2 | 0.123(10) | 0.212(56) | 0.203(71) | 0.30(21) | 0.174(44) | 0.049(80) | 0.376(64) |
| | c1 | 0.0096(91) | 0.0021(36) | 0.0098(86) | 0.018(13) | 0.0015(26) | 0.006(11) | 0.070(96) |
| | c2 | 0.070(56) | 0.071(74) | 0.014(23) | 0.22(17) | 0.17(12) | 0.114(62) | 0.28(12) |
| | d1 | * | 0.010(18) | * | * | 0.0014(23) | * | 0.0028(28) |
| | d2 | 0.006(11) | 0.0060(57) | 0.0093(27) | 0.0121(62) | 0.013(20) | 0.041(37) | 0.007(12) |

*No detectable signal was observed in the OSLD readout.

## References


[1] Christensen JB, Muñoz ID, Bassler N, Stengl C, Bossin L, Togno M, et al. Optically stimulated luminescence detectors for dosimetry and LET measurements in light ion beams. Phys Med Biol 2023;68:155001. https://doi.org/10.1088/1361-6560/acdfb0 .

[2] Stengl C, Panow K, Arbes E, Muñoz ID, Christensen JB, Neelsen C, et al. A phantom to simulate organ motion and its effect on dose distribution in carbon ion therapy for pancreatic cancer, Phys Med Biol 2023;68:245013. https://doi.org/10.1088/1361-6560/ad0902 .

[3] Haberer T, Debus J, Eickhoff H, Jäkel O, Schulz-Ertner D, Weber U. The Heidelberg Ion Therapy Center. Radiother Oncol 2004:73;186–90. https://doi.org/10.1016/S0167-8140(04)80046-X .
